# *Babesia microti* Immunoreactive Rhoptry-Associated Protein-1 Paralogs Are Ancestral Members of the Piroplasmid-Confined RAP-1 Family

**DOI:** 10.3390/pathogens10111384

**Published:** 2021-10-26

**Authors:** Reginaldo G. Bastos, Jose Thekkiniath, Choukri Ben Mamoun, Lee Fuller, Robert E. Molestina, Monica Florin-Christensen, Leonhard Schnittger, Heba F. Alzan, Carlos E. Suarez

**Affiliations:** 1 Department of Veterinary Microbiology and Pathology, College of Veterinary Medicine, Washington State University, Pullman, WA 99164, USA; heba.alzan@wsu.edu; 2 Fuller Laboratories, 1312 East Valencia Drive, Fullerton, CA 92831, USA; jose.thekkiniath@fullerlabs.net (J.T.); Lee.Fuller@fullerlabs.net (L.F.); 3 Section of Infectious Diseases, Department of Internal Medicine, Yale School of Medicine, New Haven, CT 06520, USA; choukri.benmamoun@yale.edu; 4 Protistology Laboratory, American Type Culture Collection, Manassas, VA 10801, USA; rmolestina@atcc.org; 5 Consejo Nacional de Investigaciones Científicas y Técnicas (CONICET), Buenos Aires C1033AAJ, Argentina; jacobsen.monica@inta.gob.ar (M.F.-C.); schnittger.leonhard@inta.gob.ar (L.S.); 6 Instituto de Patobiología Veterinaria, CICVyA, INTA-Castelar, Hurlingham, Buenos Aires C1033AAE, Argentina; 7 Parasitology and Animal Diseases Department, National Research Center, Dokki, Giza 12622, Egypt; 8 Tick and Tick-Borne Disease Research Unit, National Research Center, Dokki, Giza 12622, Egypt; 9 Animal Disease Research Unit, United States Department of Agricultural—Agricultural Research Service, Pullman, WA 99164, USA

**Keywords:** *Babesia microti*, BmIPA48, BMR1_03g00960, piroplasmid rhoptry-associated protein-1 (pRAP-1), human babesiosis

## Abstract

*Babesia*, *Cytauxzoon* and *Theileria* are tick-borne apicomplexan parasites of the order Piroplasmida, responsible for diseases in humans and animals. Members of the piroplasmid rhoptry-associated protein-1 (pRAP-1) family have a signature cysteine-rich domain and are important for parasite development. We propose that the closely linked *B. microti* genes annotated as BMR1_03g00947 and BMR1_03g00960 encode two paralogue pRAP-1-like proteins named BmIPA48 and Bm960. The two genes are tandemly arranged head to tail, highly expressed in blood stage parasites, syntenic to *rap-1* genes of other piroplasmids, and share large portions of an almost identical ~225 bp sequence located in their 5′ putative regulatory regions. BmIPA48 and Bm960 proteins contain a N-terminal signal peptide, share very low sequence identity (<13%) with pRAP-1 from other species, and harbor one or more transmembrane domains. Diversification of the piroplasmid-confined *prap-1* family is characterized by amplification of genes, protein domains, and a high sequence polymorphism. This suggests a functional involvement of pRAP-1 at the parasite-host interface, possibly in parasite adhesion, attachment, and/or evasion of the host immune defenses. Both BmIPA48 and Bm960 are recognized by antibodies in sera from humans infected with *B. microti* and might be promising candidates for developing novel serodiagnosis and vaccines.

## 1. Introduction

*Babesia*, *Cytauxzoon* and *Theileria* are tick-borne apicomplexan piroplasmid parasites of vertebrates that invade and reproduce asexually in erythrocytes. These parasites are a major concern to human and animal health and cause an important economic burden worldwide. *Babesia* parasites are responsible for acute and persistent hemolytic disease in several wild and domestic vertebrate species, including human. While *Theileria* parasites are transmitted transstadially by ticks, *sensu stricto* (s.s.) *Babesia* spp. are transovarially and, in some species, also transstadially, transmitted. Other piroplasmids, such as *B. microti*, are defined as *sensu lato* (s.l.) *Babesia* parasites, based on their transstadial mode of transmission and the absence of schizont stages in their life cycles [1,2,3].

Human babesiosis is an emergent worldwide zoonosis caused by several *Babesia* spp., including the s.s. *B. divergens* and the s.l. *B. microti*, the latter of which is the predominant agent in the Northeastern and Midwest regions of the US [4,5]. As for other piroplasmids, the life cycle of *B. microti* is dixenic, involving an invertebrate definitive host and a vertebrate host. In the US, the primary vertebrate host is the white-footed mouse (*Peromyscus leucopus*) and the invertebrate host is a tick of the genus *Ixodes*, such as *I. scapularis*. However, humans are accidental and dead-end hosts when bitten by infected ticks. Importantly, human-to-human transmission of *B. microti* may occur via contaminated blood transfusions [6,7]. Due to climate change and human activity, the geographic distribution of *I. scapularis*, and hence of *B. microti*, is expanding rapidly in the US [8]. In addition, the finding of vertical transmission in the white-footed mouse indicates a potentially relevant way of parasite dissemination without the participation of the tick vector [9]. The disease caused by *B. microti* in humans may vary from asymptomatic or subclinical to acute and chronic manifestations, which can be lethal in immunocompromised patients. Clinical manifestations of acute human babesiosis include fever, hemolytic anemia, acute respiratory distress and multiorgan dysfunction [10]. Because of the expansion of the tick habitat and the constant increase in cases of human babesiosis in the US, there is a need to develop vaccines and improved diagnostics against *B. microti*, which requires identification of conserved immunogenic proteins in this apicomplexan parasite.

Apicomplexan parasites, including *B. microti*, are equipped with an apical complex with at least three distinct secretory organelles known as the rhoptries, micronemes, and spherical bodies or dense granules. These organelles play an essential role in host cell invasion by the parasite [11]. Once the parasite is committed to invasion, it is quickly and actively propelled inside the target cell by the activity of an actin motor, with intervention of the cytoskeletal structures of the parasite [12]. Rhoptry proteins are probably involved in the formation of the parasitophorous vacuole (PV), a membranous structure separating the parasite from the cytoplasm of the host cell, that disappears quickly upon invasion in *Babesia* parasites [13]. Remarkably, *B. microti* also developed a mechanism for vesicle-mediated antigen export generating an interlacement of vesicles which extends from the plasma membrane of the parasite into the cytoplasm of the host erythrocyte [14]. Few rhoptry proteins have been so far identified and characterized in *Babesia* parasites. Initial studies performed mainly in *B. bovis* and *B. bigemina* were focused on the functional role of rhoptry-associated protein-1s (RAP-1s), which were later identified in all piroplasmids, including other *Babesia* spp., *Theileria* spp. and *Cytauxzoon felis* [15,16,17,18,19,20,21,22,23]. We hereby refer to these proteins as piroplasmid RAP-1s (pRAP-1s). It is possible that the function of these piroplasmid-specific proteins is needed to support unique features of the parasite life cycle, such as parasite-attachment to the erythrocyte, dissolution of the PV in *Babesia*, the zipper-mediated invasion of *Theileria*, or other events that may be related to erythrocyte invasion and egress [24,25]. The *prap-1* gene superfamily encodes the paralogs *rap-1* and RAP-1-related antigens (*rra*) in *B. bovis* [26]. Plasmodial RAP-1 shares the same denomination with pRAP-1s, but they are unrelated non-homologous proteins [27]. Since pRAP-1 proteins are highly immunogenic and can be targeted for neutralization-sensitive antibodies, they may be attractive candidates for diagnostic assays or subunit vaccines against *Babesia* and *Theileria* parasites [16,28,29,30,31,32,33,34].

The piroplasmid-specific RAP-1 family domain (PF03085) contains a characteristic motif of four cysteine (Cys) residues and a single conserved tyrosine (Tyr) residue. Other definitions of the members of this protein family are based on localization or function, which are still waiting experimental confirmation. Although the pRAP-1 proteins have been identified and annotated in genomes of *Babesia* spp. s.s., *Cytauxzoon felis*, and *Theileria* spp. parasites, they remain not fully identified in the genome of the s.l. parasite *B. microti*. A RAP putative protein (XP_021337499) was annotated in the genome of *B. microti*, but this protein, which is homologous to *Plasmodium* and *Toxoplasma* RAPs, lacks the characteristic motifs of the members of the *Babesia*/*Theileria* pRAP-1 superfamily [35]. Thus, the presence of canonical *Babesia*/*Theileria* pRAP-1 genes has yet to be reported in *B. microti*. We hypothesized that, like *Babesia* and *Theileria* parasites, the genome of *B. microti* also includes genes encoding for pRAP-1 or pRAP-1-like proteins. Furthermore, because of the relatively distant phylogenetic relationship of *B. microti* with piroplasmid parasites such as *Babesia* s.s. and *Theileria* s.s. [1], we propose that the pRAP-1-like proteins encoded by *B. microti* may have diverged dramatically from the pRAP-1 molecules expressed in other piroplasmids, resulting in a low non-significant sequence identity, but conservation of important structural features. Indeed, neither a common BLASTp nor a Pfam search resulted in hit or domain report, respectively. Therefore, we carried out alternative search strategies on the *B. microti* genome based on the previously detected conserved synteny in the genome regions of piroplasmid parasites where the *prap-1* loci are encoded and found two head-to-tail oriented linked genes, BMR1_03g00947 and BMR1_03g00960, encoding for proteins with structural characteristics that are compatible with the pRAP-1 molecules. Although the database searches did not result in hits, synteny analysis and the presence of highly conserved amino acid residues of structural importance organized as the Cys-rich domains of the pRAP-1s proteins strongly suggest that the presented two genes encode for pRAP-1 homologs in *B. microti*. Since these putative *B. microti* pRAP-1 proteins lack significant sequence identity with pRAP-1 domains of other pRAP-1s, we designated them pRAP-1-like proteins. For the aforementioned reasons, *B. microti* pRAP-1-like proteins have previously remained unnoticed, though these proteins have been identified and shown to be expressed in *B. microti* merozoites [35,36].

## 2. Results

### 2.1. Two Tandemly Arranged RAP-1 Syntenic Genes of B. microti Encode Proteins Containing Non-Canonical Piroplasmid RAP-1 Cys-Rich Domains

**Figure 1:**
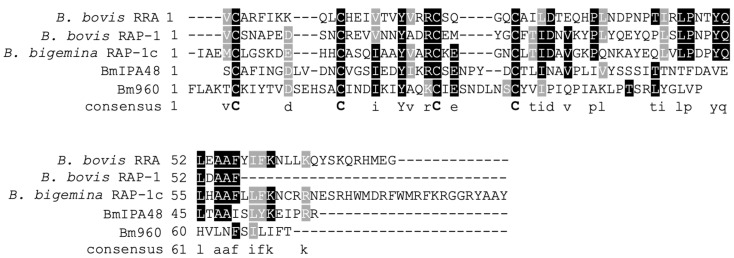
Representative figure of the comparisons performed with the pRAP-1 Cys-rich motif among babesial rRAP-1 proteins and the newly identified *B. microti* putative pRAP-1. The comparisons include the Cys-rich regions of *B. bovis* RRA, *B. bovis* RAP-1, *B. bigemina* RAP-1c, and the *B. microti* RAP-1- like proteins BmIPA48 and Bm960.

**Figure 2:**
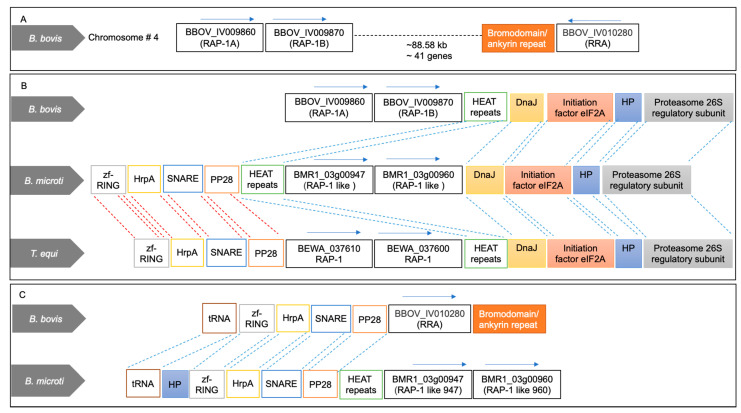
Synteny map of the *rap-1* locus of *T. equi*, putative *rap-1 B. microti*, and typical s.s. *B. bovis*. (**A**) Structure of the *prap-1* and *rra* genes localized in the chromosome 4 of *B. bovis*. (**B**) Conserved synteny among the *rap-1* loci of *B. bovis* and *T. equi* and the BMR1_03g00960 (BmIPA48) and BMR1_03g00947 (Bm960) genes of *B. microti*, that encode for proteins containing the typical Cys-rich region of the pRAP-1 proteins. (**C**) Conserved synteny among the *B. bovis rra* and the *B. microti* BMR1_03g00960 and BMR1_03g00947 genes.

The piroplasmid RAP-1 proteins contain a characteristic Cys-rich domain, signal peptide, and other short conserved sequence motifs. The salient features of some typical pRAP-1 and RRA representatives of this family are schematized in Figure S1. In this study, we first searched the predicted proteome of the *B. microti* R1 strain for the identification of proteins containing pRAP-1 Cys motifs using Delta-Blast analysis against a query of the *B. bigemina* RAP-1c Cys-rich domain (CLGSKDEHHCASQIAAYVARCKE), also typical for the pRAP-1s of *B. bovis* (Figure 1). This search revealed a hit with the hypothetical protein encoded by gene BMR1_03g00960, here referred to as Bm960 (Figure 1). This finding prompted us to investigate the corresponding gene locus for the presence of other *rap-1* related genes and for synteny with *B. bovis*, *B. bigemina* and *T. equi rap-1* loci. Sequence analysis revealed that the BMR1_03g00947 gene, reffered to as BmIPA48 (Figure S1), located immediately next to Bm960, and separated by an 800-bp intergenic region, encodes for a protein also containing a similar RAP-1-like Cys-rich region, including a key conserved Tyr residue in its amino terminal (Figure 1, Figure 2B,C and Figure S2).

**Figure 3:**
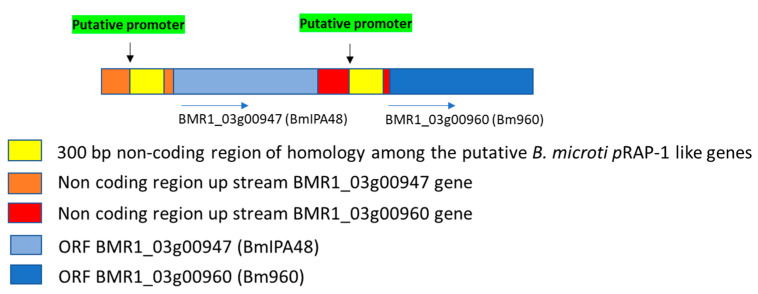
Schematic representation of the locus encoding for the *B. microti* RAP-1 putative proteins BmIPA48 and Bm960. A ~300 bp region upstream the two ORFs is repeated (yellow boxes).

**Figure 4:**
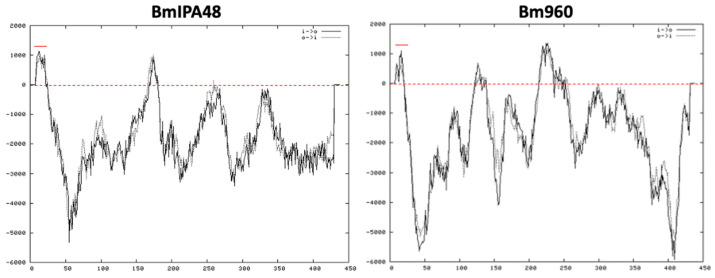
Predicted secondary structure of the pRAP-1-like proteins BmIPA48 and Bm960 using the Program TMpred. Predicted location of signal peptide (SP) is marked with a red bar. A dashed red line marks the boundary between predicted hydrophilic and hydrophobic transmembrane regions of the proteins.

As shown in Figure 2A, the chromosome 4 of *B. bovis* contains two identical head-to-tail arranged *prap-1* genes, and a single gene encoding for the RRA protein. These two loci are separated by a 88.5 kb intervening region containing ~41 genes. A comparison between the rap-1 loci of *B. bovis* and *T. equi* with the *B. microti* locus containing BmIPA48 and Bm960 genes is shown in Figure 2B. This illustration shows full synteny in the 5′ and 3′ ends of the *B. microti* BmIPA48 and Bm960 gene locus and the *prap-1* locus of *T. equi.* In Figure 2C we illustrate partial synteny of the 3′ end of the *B. microti* genes and the rra locus of *B. bovis*. Besides the presence of the unique Cys-rich regions, there was no significant sequence similarity among the putative pRAP-1 proteins encoded by the BmIPA48 and Bm960 genes (Figure S2). However, the alignment shows conserved Cys, Tyr, and other typical residues of the pRAP-1 proteins in the NT-region of the molecules, as well as other short amino acid motifs (Figure S2). The protein encoded by gene BMR1_ BmIPA48 also contains a series of tandem repeats in its C-terminal region, a feature that is shared with the *B. bovis* RAP-1 proteins (Figure 3 and Figure S3). Strikingly, sequence analysis of the non-coding regions immediately upstream of genes BmIPA48 and Bm960 revealed conservation of a 300-bp sequence (Figure 3 and Figure S4), suggesting that expression of these two proteins might be coordinated, despite their non-relatedness in sequence. In addtion, secondary structure sequence analysis performed in silico using TMpred suggests that BmIPA48 contains a signal peptide (aa 4–24) and a putative transmembrane (TM) region (aa 164–186) (Figure 4). Since no TM domains were previously reported in this protein, the prediction was confirmed using the alternative algorithm Phobius, which also showed the presence of a TM domain in the same region (Figure S6). Bm960 protein also contains a predicted signal peptide, two TM domains, and lack a predicted GPI anchor attachment site (Figure 4). Collectively, these features are fully consistent with expression on the surface of the parasite, as previously predicted [35].

### 2.2. Significance of Synteny Relationships among rap-1 and rra Genes of Babesia and Theileria

After identifying BmIPA48 and Bm960 as two *B. microti* encoded proteins containing non-canonical piroplasmid RAP-1 Cys-rich domains, we perfomed synteny analysis of these genes. Results showed a remarkable synteny conservation of the *rap-1* locus in piroplasmids (Figure 2). The BmIPA48 and Bm960 locus is in close vicinity with 3 genes that are also in the neighborhood of the *rap-1* genes in *B. bovis* and *Theileria* spp. (Figure 2). Moreover, one of these neighboring genes, encoding for the platelet-derived GF associated protein, is also associated with the locus of the *rra* gene of *B. bovis*. The schematic representation of the *rap-1* loci of *B. microti*, *Theileria* and *Babesia* s.s. in Figure 2 suggests the occurrence of a mechanism of genomic rearrangement in a chromosome of an ancestral *Babesia* organism that resulted in the insertion of an intervening region (~88 kb) encoding 41 genes in the case of *B. bovis* (Figure 2A).

### 2.3. Phylogeny of Piroplasmid RAP-1 Proteins Recapitulates Piroplasmid Phylogeny

**Figure 5:**
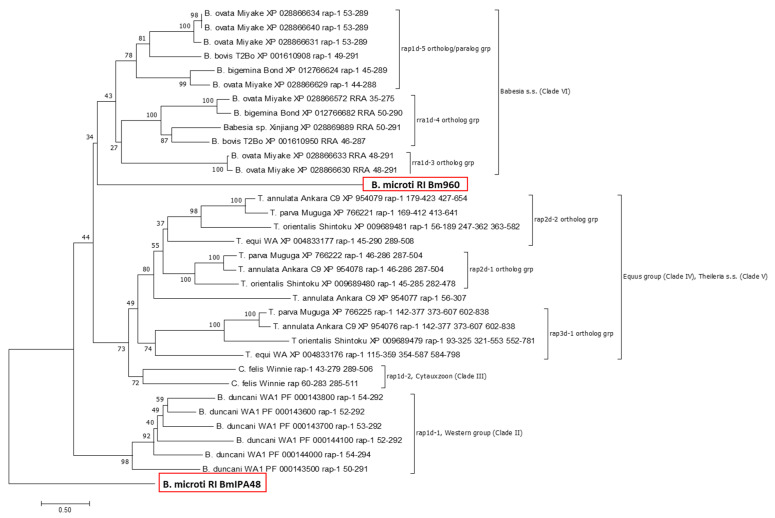
Phylogenetic neighbor joining tree inferred using amino acid sequences of pRAP-1 from the reference genomes of s.s. *Babesia* (Clade VI), s.s. *Theileria* (Clade V), *T. equi* (Clade IV), *C. felis* (Clade III), and *B. duncani* (Clade II) and the *B. microti* RAP-1 proteins BmIPA48 and Bm960 (bold fonts, red boxes). Bootstrap values of 1000 replicates are shown next to the branches. BmIPA48 is used as outgroup. The scale gives the evolutionary distance used to construct the tree.

Next, we inferred on the phylogenetic relationship between amino acid sequences of pRAP-1-like BmIPA48 and Bm960 (Clade I, *B. microti*-group: *B. microti* RI) with that of pRAP-1 proteins encoded in available reference genomes of piroplasmid species belonging to Clade II (Western clade: *B. duncani* WA), Clade III (Cytauxzoon: *C. felis* Winnie), Clade IV (Equus group: *T. equi* WA1), Clade V (*Theileria* s.s: *T. annulata* Ankara C9, *T. parva* Muguga, and *T. orientalis* Shintoku), and Clade VI (*Babesia* s.s.: *B. bovis* T2Bo, *B. ovata* Miyake, *B. bigemina* Bond, *Babesia* sp. Xinjiang) (Clades as defined by Schnittger et al. 2012 [1], Jalovecka et al. 2019 [3]) (Figure 5). Based on the assumption that *B. microti* is distantly related to other piroplasmid species, the tree was rooted using *B. microti* RI RAP-1-like BmIPA48 as an outgroup. As can be seen in Figure 5, the constructed pRAP-1 protein tree recapitulates phylogenetic lineages of piroplasmids as previously reported [1]. However, Bm960 places with a low bootstrap (bs: 34) as sister taxon to remaining *Babesia* s.s. pRAP-1 proteins due to its low sequence identity with other pRAP-1 proteins.

Because of the expansion in the number of pRAP-1 domains in *Theileria* and *Cytauxzoon*, here we propose a new nomenclature for this gene family, which is based on the number of pRAP-1 domains in encoded proteins and shown in Figure 3 and Figure 5. Thus, *rap1d*, *rap2d*, and *rap3d* genes encode for pRAP-1 proteins that comprise of a single (as seen in *B. microti*, Clade I; *B. duncani*, Clade V; and *Babesia* s.s., Clade VI), a tandemly repeated (as seen in *Cytauxzoon*, Clade III; *T. equi*, Clade IV, and *Theileria* s.s. Clade V), or tandemly triplicated pRAP-1 domains (*T. equi*, Clade IV, and *Theileria* s.s. Clade V), respectively (Table 1). Furthermore, an additional number refers to the placement into different orthologous groups within each piroplasmid phylogenetic lineage (Figure 5).

Interestingly, two orthologous groups of *Babesia* s.s. (*rap1d-3* and *rap1d-4*) correspond with chromosomal rearrangements that have resulted in the generation of RRA proteins, which, although their pRAP-1 domain is complete, are shortened at their C-terminal end and are only found in *Babesia* s.s. (Figure 1). As shown in Figure 2, *Babesia* parasites contain two or more copies of pRAP-1 and a single additional RRA located ~40–80 kb from the pRAP-1 locus, separated by the insertion of an intervening sequence (Figure 2A). Results show that this is not the case for *Theileria* parasites, which did not undergo the splitting of the *rap-1* locus due to chromosome rearrangements and thus, lack *rra* genes (Figure 2A).

### 2.4. BmIPA48 and Bm960 Are Immunogenic during Infection in Humans

**Figure 6:**
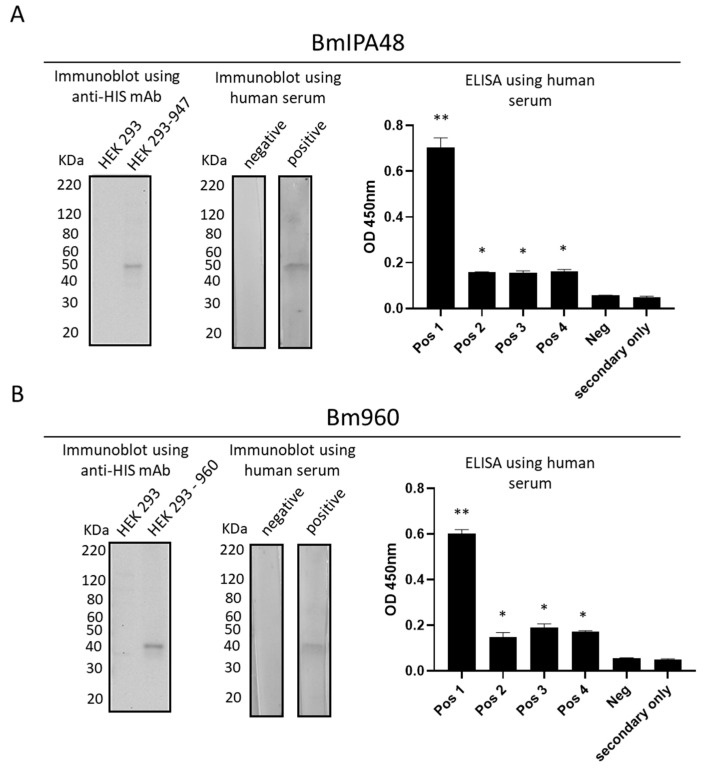
Immunogenicity of BmIPA48 and Bm960 in *B. microti*-infected humans. Expression of the recombinant BmIPA48 and Bm960 containing a HIS-tag in the immunoblots was demonstrated using an anti-HIS monoclonal antibody (panels (**A**,**B**), respectively). A control lysate of cells not expressing the recombinant protein was included as a negative control (HEK 293). Immunoblots were incubated with human *B. microti* positive and negative sera. ELISAs were performed with four positive (Pos 1, Pos 2, Pos 3, and Pos4) and one negative (Neg) human serum samples were tested. A control sample incubated only with secondary anti-human IgG serum (secondary only) was also included in the ELISA analysis. ** *p* < 0.001. * *p* < 0.01.

A previous study identified BmIPA48 and Bm960 proteins as possible biomarkers of acute infection by using a combination of nanoparticle harvesting technology and mass spectrometry on blood derived from *B. microti* infected hamsters [37]. Even though the antigenicity of Bm960 was not investigated in detail, the protein was not recognized by global antibody screening in rodent models. Bm960 was found to be highly polymorphic among strains [35], and to be present in the plasma of infected hamsters [36], confirming that it is a component of the secretome of the parasite. So far, the immunogenicity of BmIPA48 and Bm960 proteins has remained unknown in *B. microti*-infected humans. We then investigated whether sera from B. microti infected humans contain antibodies that recognize these two proteins. To this end, we expressed and purified recombinant HIS-tagged truncated forms of BmIPA48 and Bm960 proteins. The recombinant proteins were analyzed in ELISA and immunoblot using previously characterized sera from *B. microti*-infected humans (Figure 6). Antibodies from four *B. microti*-infected individuals recognized BmIPA48 and Bm960 in ELISA. Immunoblot analysis showed that antibodies from infected humans reacted with a product of expected size of BmIPA48 and Bm960 recombinant proteins, as recognized by control anti-HIS monoclonal antibody (Figure 6).

## 3. Discussion

In this study we identified two pRAP-1 in *B. microti*, named BmIPA48 and Bm960. Previous work indicated that BmIPA48 and Bm960 are highly expressed by *B. microti* merozoites and present in the parasite secretome [35]. The BMR1_03g00947 protein was previously identified erroneously as an orthologue of the *P. falciparum* gene PF3D7_1324300, and given the designation of BmIPA48, which is kept in the present study to avoid confusion [35,36]. However, alignment of PF3D7_1324300 and BmIPA48 reveals that their similarity is limited mainly to the glycine residues located in the tandem repeat regions of the two proteins (Figure S5). The tandem repeat region in PF3D7_1324300 is not pan-conserved among *Plasmodium* proteins, suggesting that it might lack functional or structural relevance, but instead, it may work as a decoy for the immune system of the host. This observation also suggests that the repeat segment may be a result of convergent evolution, and thus BmIPA48 might not be a true orthologue of PF3D7_1324300. Also, BmIPA48 contains non-synonymous polymorphisms, including a variable microsatellite region, that is highly antigenic and secreted, as part of tubes of vesicles during infection in mice [14,35]. In addition, electron microscopy analysis demonstrated that BmIPA48 is localized inside lipid-rich vesicles, which is consistent with their exclusive association with a membrane fraction. Also, IFA shows association of BmIPA48 with the cytoplasm of infected erythrocytes [37]. The presence of previously unnoticed TM domains in BmIPA48 is compatible with the association and export of this protein via lipid-rich vesicles to the cytoplasm of host erythrocytes and eventually to the outside of the host cell, as previously reported [14].

Considering that random gene location associations among four gene loci are highly unlikely in genomes larger than 8 Mb as those of *Babesia* and *Theileria* parasites, the data strongly suggest that the BmIPA48 and Bm960 genes are positional equivalents of the *Babesia-Theileria rap-1* genes. The biological significance of conserved gene synteny remains undefined. However, co-localization of genes may be important in epigenetic mechanisms and may influence the topology of the chromatin, which in turn can heavily influence coordinated gene expression and gene evolution. It is possible that the presence of syntenic genes results in the advantages of sharing regulatory mechanisms [38]. Sequence analysis of the non-coding regions immediately upstream of BmIPA48 and Bm960 showed conservation of a 300-bp sequence, suggesting a potential coordinated expression of these genes. Notably, a similar feature was found in other *Babesia* tandemly arranged and closely related or identical gene pairs or triplets, such as the *B. bovis* *rap-1s*, *msa-2s*, and *ef-1α*, that share common 5′ untranslated regions [15,39,40,41].

A remarkable synteny conservation of the *rap-1* locus in piroplasmids is shown by the data in our study (Figure 2). In addition, the insertion of an intervening region may have resulted in the splitting of the original *rap-1* locus, favoring independent gene evolution of the two identical copies of *rap-1* and *rra* genes [15,26,42]. A similar gene organization is found in *B.* *bigemina* with a *rra* gene separated by a similar large intervening region from a highly diversified and complex *rap-1* locus [17]. Interestingly, the intervening region between *rra* and *rap-1* in *Babesia* parasites is located ~100 kb upstream in the same chromosome in the *B. microti*, as well as in the *T. equi* genomes. However, all the *rap-1* genes are located together in a single cluster in this group of organisms, which also lack *rra* genes. Altogether, comparative analysis of the locus encoding *B. microti* BmIPA48 and Bm960 proteins with the loci of *Babesia* and *Theileria rap-1* genes provides interesting insights on the synteny and the evolution of the genome of these parasites.

Results from the phylogenetic analysis supports the notion that Bm960 cannot be defined as pRAP-1 based on sequence identity, but only due to structural conservations and synteny. Importantly, it can also be concluded from the phylogenetic tree that pRAP-1 is a relatively complex highly polymorphic protein family that underwent multiple duplications into large gene families of paralogs, tandem duplications and triplications of the pRAP-1 cys-rich domain, and a substantial nucleotide diversification, resulting in the existence of multiple highly polymorphic pRAP-1 domains. Thus, this protein family displays a considerable complexity, typically observed for molecules that play a pivotal functional role in the parasite-host interface, such as adhesion, attachment, and invasion, or the interaction with the host immune defense [43]. We hypothesize that the generation of diversification of pRAP-1 proteins is driven by a strong positive selection to optimize adhesion and attachment to their different hosts, as is required for the evolution of parasite host-specificity. The different copy number of pRAP-1 domains in a single protein may represent an adaptation strategy to different hosts and life cycles, enabling the parasites to invade different host species and cells. *C. felis* has two tandemly arranged pRAP-1 proteins containing canonical domains, while most *Theileria* has pRAP-1 proteins with tandemly duplicated or triplicated pRAP-1 domains. Both the *C. felis* and *Theileria* pRAP-1 domains contain 4 conserved Cys residues and a single conserved Tyr residue, as originally described in *Babesia* [15,17,26]. Considering that certain pRAP-1 features correspond with the phylogenetic classification of piroplasmid species, this finding may be exploited for the development of specific diagnostic tests. Furthermore, pRAP-1 proteins with a duplicated and/or triplicated domain architecture specify the piroplasmid lineages *Cytauxzoon*, *Theileria equi*, and *Theileria* s.s. and contrast with those that encode exclusively single-domain pRAP-1s, such as *Babesia* s.s. and *Babesia* s.l. [1].

*B. microti* contains two *prap-1*-like genes located as a single cluster in the region of the genome where s.s. *Babesia* and *Theileria* organisms contain their *prap-1* genes. These two genes have a fully conserved synteny and identical flanking genes as *Theileria* parasites, as shown in Figure 2A. This implies that the aforementioned genome rearrangements resulted in an independent evolution of RRA encoding genes, which likely occurred after *Babesia* organisms emerged as separate species from a common *Babesia* and *Theileria* ancestor. This notion is further supported by the observation that all proteins segregating into the *rap1d-3* and the *rap1d-4* orthologous groups represent RRA proteins since, although they contain a complete pRAP-1 domain, are shortened at the C-terminal end (Figure 1). This strongly suggests that the ancient RRA protein has lost its C-terminal partly due to chromosomal rearrangement and places this event before the diversification of the RRA proteins.

Considering that the antigenicity of BmIPA48 and Bm960 was not previously investigated [35,36,37,39], here we examined sera from *B. microti*-infected humans for the presence of antibodies against these proteins. Collectively, results of ELISA and immunoblot indicate that BmIPA48 and Bm960 are immunogenic during infection in humans, and thus should be considered for further testing as possible candidates for serological diagnosis of human babesiosis caused by *B. microti*. In addition, because of their previously established high degree of expression, surface localization, conservation, and immunogenicity, BmIPA48 and Bm960 proteins might also be promising candidates for the development of vaccines that may prevent human babesiosis.

## 4. Materials and Methods

### 4.1. Expression of Recombinant B. microti pRAP-1 like Proteins

The predicted proteins encoded by *B. microti* BMR1_03g00947 and BMR1_03g00960 (GenBank accession numbers: XP_021338473 and XP_021338474, respectively), here referred to as BmIPA48 and Bm960, respectively, were analyzed by the Kyte-Doolittle scale for the presence of hydrophobic regions as previously described [44]. As a result, 105 nt and 84 nt-long fragments located at the 5′ end of BMR1_03g00947 and BMR1_03g00960, correspondingly, encoding hydrophobic peptide segments, were excluded from the cloning and protein expression experiments described in this work. The resulting nucleotide sequences were codon-optimized for mammalian cell expression, synthesized by GenArt Gene Synthesis (Thermo Fisher Scientific, Waltham, MA, USA) and cloned into pcDNA3.4. Recombinant plasmids containing either truncated BMR1_03g00947 (pcDNA3.4/947) or truncated BMR1_03g00960 (pcDNA3.4/960) were fully sequenced to confirm the presence of the target genes in frame with the cytomegalovirus promoter (data not shown). Subsequently, HEK 293 cells were transiently transfected with either pcDNA3.4/947 or pcDNA3.4/960 using polyethylenimine, as described elsewhere [45]. Expression of the recombinant truncated proteins (BmIPA48tr and Bm960tr) was confirmed by immunoblot using the anti-6xHis monoclonal antibody (clone AD1.1.10) (Bio-Rad, Hercules, CA, USA). Recombinant BmIPA48tr and Bm960tr were purified using the HisPur™ Cobalt Purification Kit following the manufacturer’s protocol (Thermo Fisher Scientific). After purification, the recombinant proteins were dialyzed using the Slide-A-Lyzer™ Dialysis cassettes (Thermo Fisher Scientific) and stored at −80 °C until use for ELISA and immunoblot.

### 4.2. Human Serum Samples

Unidentified human patient serum samples were submitted to Fuller Laboratories from Labcorp, NC for anti-*B. microti* IgG determination. No clinical data were provided for any of the specimens.

### 4.3. ELISA Procedure

Antigen dilution was performed by mixing 8 µL of recombinant BmIPA48 antigen (approx. 0.5 μg/μL) in 500 µL PBS buffer followed by two-fold dilutions 1:2, 1:4 and 1:8. For Bm960, 4 µL of the recombinant antigen (approx. 1 μg/μL) was mixed in 500 µL PBS buffer, and then diluted two-fold 1:2, 1:4 and 1:8. Two neighboring strips of the ELISA plate were coated with 100 µL/well of 1:4 and 1:8 antigen dilutions. The antigen-coated plates were incubated at room temperature (23–25 °C) overnight, then back coated by adding 100 μL/well WellChampion (Microwell Plate Blocker/Stabilizer, Kementec, Copenhagen, Denmark) to each well for 5–10 min. Plates were then decanted and allowed to dry overnight in a dark low-humidity room before use. Negative serum control was obtained from a non-reactive unidentified human patient, which tested negative in confirmatory IFA analysis. Positive controls (*n* = 4) corresponded to anti-*B. microti* IgG and IgM reactive unidentified human sera with IFA endpoint titers > 1:1024 (cat. BMG-120, Fuller Laboratories, Fullerton, CA, USA). IFA testing utilized both hamster in vivo and human type O in vitro antigen (US 10,087,412 B2 patent). All sera were diluted 1:100 in sample diluent (PBS/2 mg/mL bovine serum albumin/0.1% Tween-20). One hundred µL aliquots of diluted sera were added to ELISA plate microwells. Two rows of microwells were filled with sample diluent and were used for secondary antibody controls. Plates were covered to minimize evaporation and incubated for 60 min at room temperature. Then, plates were washed four times with wash buffer (PBS/0.1% Tween-20). One hundred µL of a working dilution of anti-human IgG (γ-chain-specific)-horseradish peroxidase (HRP) conjugate (SFG-1X, Fuller Labs) were added to each well, and the plate was covered and incubated for 30 min at RT in the dark. Microwells were washed as above and 100 µL TMB substrate was added to each well. Reactions were allowed to proceed for exactly 10 min in the dark and interrupted by adding 100 µL Stop solution (0.36 N sulfuric acid). Absorbance at 450 nm was read in a microplate reader (MultiSkan MCC/340, Titertek, Pforzheim, Germany). Absorbance values of *B. microti* positive and negative sera were compared by Student’s *t*-test using Prism version 6 (GraphPad Software, San Diego, CA, USA).

### 4.4. Immunoblot Analysis

For human serum analysis, aliquots (45 μL) of recombinant BmIPA48 and Bm960 antigens were separated using 10% Mini-PROTEAN^®^ TGX™ Precast Protein Gels (Bio-Rad, Cat #4561034) and transferred to PVDF membranes. Membranes were blocked with 5% milk, cut into strips, and individually incubated overnight at 4 °C with *B. microti*-positive or negative patient sera, in a 1:250 dilution. Membranes were then washed with PBS/0.1% Tween-20 and incubated for 1 h with HRP-conjugated secondary antibody (1:10,000 dilution). Following additional washings, membranes were incubated with Opti-4CN substrate diluted in 1-part Opti-4CN diluent and 9 parts distilled water (Bio-Rad, Cat# 1708235) for 5–30 min until the desired the signal was obtained.

### 4.5. Bioinformatic Analysis

Secondary sequence analysis was performed using TMpred Server (vital-it.ch, accessed on 1 August 2021) and Phobius (phobius.sbc.su.se, accessed on 1 August 2021). Prediction of GPI anchor signals was carried out using PredGPI (gpcr.biocomp.unibo.it/predgpi/, accessed on 1 August 2021). Synteny studies were carried out by exploring the Piroplasma DB database (piroplasmadb.org/piro/app, accessed on 1 August 2021).

### 4.6. Phylogenetic Analysis

The amino acid sequence of *B. bovis* T2Bo RAP-1 (XP_001610908) was used in a BLASTp search, adjusting parameter settings to piroplasmid sequences (taxid:5863) and reference proteins to identify homologs in completely sequenced genomes of piroplasmid species. The genomes analyzed included *B. bigemina* strain Bond [46], *B. bovis* strain T2Bo [47], *B. ovata* strain Miyake [48], *B. microti* strain RI [35], *C. felis* strain Winnie [49], *T. annulata* strain Ankara [50], *T. equi* strain WA [51], *T. orientalis* strain Shintoku [52], and *T. parva* strain Muguga [53]. In addition, RAP-1 sequences of *B. duncani* were retrieved by courtesy from yet public unavailable genomes (*B. duncani* strain WA1: Choukri Ben Mamoun, Yale School of Medicine, New Haven, CT, USA). Finally, BmIPA48 and Bm960 were identified by delta Blast using a RAP-1 region containing 4 conserved Cys, as described before.

Altogether 34 amino acid sequences were aligned by Muscle (www.ebi.ac.uk/Tools/msa/muscle/, accessed on 30 July 2021). In order to estimate evolutionary distances, the JTT+G (G = 5.93) was determined as best model by BIC criteria and applied [54]. After eliminating all positions with gaps and missing data, the remaining 212 positions were used for estimation of a neighbor joining tree [55]. The phylogenetic analysis was carried out using MEGA7 [56].

## 5. Conclusions

Findings in this study suggest that *rap-1* genes appeared early in the evolution of piroplasmid parasites, implying that expression of *prap-1* and *prap-1*-like genes is required for sustaining the life cycle of these organisms. Two tandemly arranged genes separated by an 800 bp intergenic region that includes a highly conserved putative promoter region are located in a region of the *B. microti* genome with strong synteny to the *prap-1* locus of *Babesia* and *Theileria* parasites. The organization of these two *prap-1*-like *B. microti* genes is reminiscent of the organization of the *prap-1* locus in *B. bovis* [15]. This feature, together with the presence of a single Cys-rich pRAP-1 motif in the encoded proteins resembles pRAP-1/RRA proteins of *Babesia*, rather than *Theileria*, parasites, but with identical synteny to *Theileria* parasites. The presence of a shared 300-bp region in the putative regulatory DNA regions suggests that the expression of these genes might be co-regulated. Both *B. microti* proteins contain TM domains and signal peptides, which is consistent with extracellular vesicle localization. Previous work showed that Bm960 is secreted into the sera of infected mice [35] and that BmIPA48 is strongly immunogenic in infected hamsters [14,36]. The gene structure comparison and phylogenetic analysis of the *prap-1* locus among distinct piroplasmid parasites allowed valuable insights on the genetic mechanisms involved in the evolution of the members of this piroplasmid-confined gene family. Importantly, this work also confirmed that antibodies in *B. microti*-infected humans recognized the recombinant forms of both proteins, so their potential as candidates for diagnostic assays and vaccines should be further explored.

## Figures and Tables

**Table 1:** Correlation of *rap* domain architecture and number of *rap-1* paralogs with phylogenetic classification of piroplasmids.

Species (Reference Genome)	Clade (Sensu Schnittger et al. 2012)	*rap-1* Domain Architectures	Number of *rap-1* Paralogs	Nomenclature (Proposed)
*B. microti*	I (*B. microti*-group)	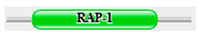	2×	*rap1d*-like
*B. duncani*	II (Western group	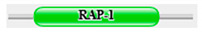	6×	*rap1d-1*
*C. felis*	III (*Cytauxzoon*)	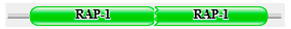	2×	*rap1d-2*
*T. e* *qui*	IV (Equus group)	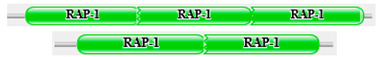	1× 1×	*rap2d-1* *rap3d-1*
*T. annulata* *T. parva* *T. orientalis*	V (*Theileria* s.s.)	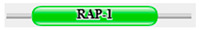 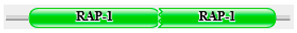 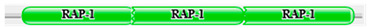	Ta:1×, Tp:1×,To:0 Ta:2×,Tp:2×,To:2× Ta:1×, Tp:1×,To:2×	*rap2d-2* *rap2d-1* *rap3d-1*
*B. bovis* *B. bigemina* *B. ovata* *Babesia* sp. Xinjiang	VI (*Babesia* s.s.)	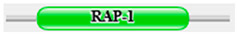	3× 2× 8× 1×	*rap1d-3* *rap1d-4* *rap1d-5*

## Data Availability

Not applicable.

## Supplementary Materials

The following are available online at https://www.mdpi.com/article/10.3390/pathogens10111384/s1, Figure S1. Schematic representation of the structural features of RAP-1 and RRA proteins, Figure S2. Amino acid sequence alignment between the BmIPA48 and Bm960 RAP-1 like proteins, Figure S3. Amino acid sequence of the Bm947 protein, Figure S4. Schematic representation of the 300 bp region of homology among the *B. microti* RAP-1 like genes BMR1_03g00947 (947) and BMR1_03g00960 (960), Figure S5. Amino acid alignment between BmIPA48 (Bm) and PF3D7_1324300 (Pf), and Figure S6. Secondary structure prediction of BmIPA48using the software Phoebius.
