# Supplementary material for: Babesia microti Immunoreactive Rhoptry-Associated Protein-1 Paralogs Are Ancestral Members of the Piroplasmid-Confined RAP-1 Family
Source: Pathogens. 2021 Oct 26;10(11):1384. doi: 10.3390/pathogens10111384 (PMC8624774; doi:10.3390/pathogens10111384)
Supplement: Supplementary file 1 [file pathogens-10-01384-s001.zip › pathogens-1406179-SM.pdf]

Supplemental Figures

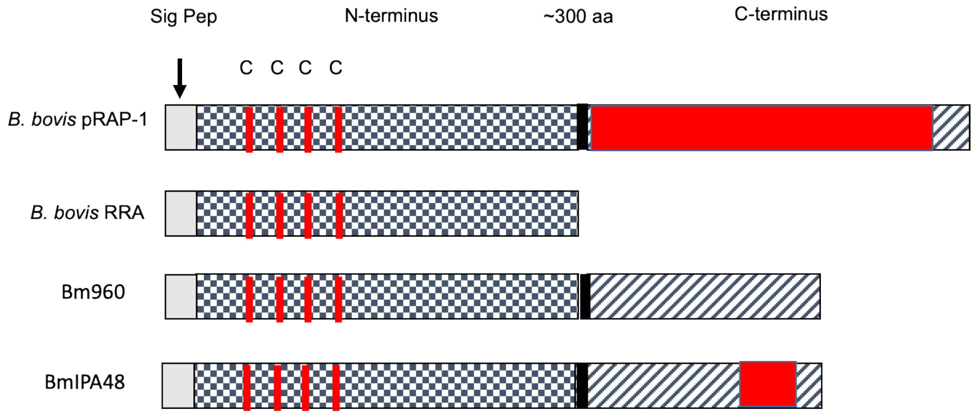

Figure S1. Schematic representation of the structural features of RAP-1 and RRA proteins. The relative location of the typical conserved Cys residues of the RAP-1 proteins is marked in red. The solid red box represents the repetitive region in the RAP-1 proteins of *B. bovis* and the RAP-1 putative *B. microti* BmIPA48 and Bm960 proteins. (The proteins are not represented in scale).

```
repeats       1 MRGMFSNKWMSFVCFSILFVALKSDLEYVSALKLLRAPPQTSLFLEKLIDDGSDIPKDPI
960           1 ------------------------------------------------------------
consensus     1 mrgmfsnkwmsfvcfsilfvalksdleyvsalkllrappqtslflekliddgsdipkdpi

repeats      61 DTDKEESQSSLFKFNLNLFNKKSIWEADEKFVITLAKSRLNVILAQKLDKFLAKTCKIYT
960           1 ---------------MSFFATE------------LIK------DKEQLLRFTNKSCAFIN
consensus    61 dtdkeesqsslfkfnl  F   siweadekfvitL Ksrlnvi    L kF  KtC

repeats     121 VDSEHSACINDIKIYAQKCIESNDLNSCYVIPIQPIAKLPTSR---LYGLVPHVLNFSIL
960          28 -GDLVDNCVGSIEDYIKRCSENP--YDCTLINAVPLIVYSSSITTNTFDAVE--LTAAIS
consensus   121 v      Ci  I  Y  kC E  dl  C vI   Pi    tS ttn y  V hvL   I

repeats     178 IFTNIRSNLDRYYIDGSKDWFSHIFMRLKRFFGIRNKHSYFSDNRLMNKIFSRTS-----
960          83 LYKEIPRRRG-FF-N--RKRITNILIRI---ITLGMYNNSYDDQTLINSLTHKLKSGIYY
consensus   181 if  l     ryyi gsk   s I mRlkrf  i      f Dn LmN i  r  sgiyy

repeats     233 TTEGPDRS--------------DSLLSN----------------YIKFGAIEYAILLNTR
960         136 KHYSPGNEQFAFSQLQLLFIGVESLTSSFIIFYRMLTLSIFDSFYSKFGISS-PIISN--
consensus   241   f P   qfafsqlqllfigvdSL S fiifyrmltlsifdsfY KFG   y Il Ntr

repeats     263 SNLVKMILSSFAHIKFVRKRLYKFYTNKWKSIEGLVTRGHLKPVDLSN--------NPIS
960         193 -KGFRLYVSYFKHMKKIGRTLVSVSKSLFKDADFFTVSGVIKEVYISLPNYISILSPSLD
consensus   301 s   km lS F HiK v k L       wK  e     G lK V lS pnyisils  i

repeats     315 DNIFKYFGKFSNNTNLSNAIA-GAFLDHYKSLFSNSTDVNGEGSSGEGPSGEGFN-----
960         252 EQQSKMFTALFASTIHNAAIEVSEIVPKL--IVLDSSTQNAIDSICKNTSGEKLSLFLCL
consensus   361 dn  K F      T    AI v   l h ksl   St  Ng  S     SGE   lflcl

repeats     369 -GEGS-----SGEGPSGEGFNGE-GFDGEGPSGEGPSGEGFNGEGFNGEGLNGEGPSGEG
960         310 RGRRTRNDLKKNTSPFEYTLIKAISKAEERQSRHQGKRRAFRDIGK--NALSGIVGFFRN
consensus   421 rG  srndlk    P        i    E  S       gF   G ng gL G

repeats     422 PSGEGLNEWNGLMNGTA
960         368 PLGKREQ----------
consensus   481 P G   newnglmngta
```

Figure S2. Amino acid sequence alignment between the BmIPA48 and Bm960 RAP-1 like proteins. Note the alignments of the 4 conserved Cys and Tyr residues at the NT-end of the proteins.

BmIPA48

mrgmfsnkwmsfvcfsilfvalksdleyvsalkllrappqtslflekliddgsdipkdpidtdkeesqs
slfkfnlnlfnkksiweadekfvitlaksrlnvilaqkldkflaktckiytvdsehsacindikiyaqkcie
sndlnscyvipiqpiaklptsrlyglvphvlnfsiliftnlrsnldryyidgskdwfshifmrlkrffgirnk
hsyfsdnrlmnkifsrtsttfgpdrsdsllsnyikfgaieyaillntrsnlvkmilssfahikfvrkrlykfyt
nkwksieglvtrghlkpvdlsnnpisdnifkyfgkfsnntnlsnaiagafldhykslfsnstdvngegs
sgegpsgegfngegssgegpsgegfngegfdgegpsgegpsgegfngegfngeglngegpsge
gpsgeglnewnglmngta

Figure S3. Amino acid sequence of the Bm947 protein. The region with repeats is marked in red font.

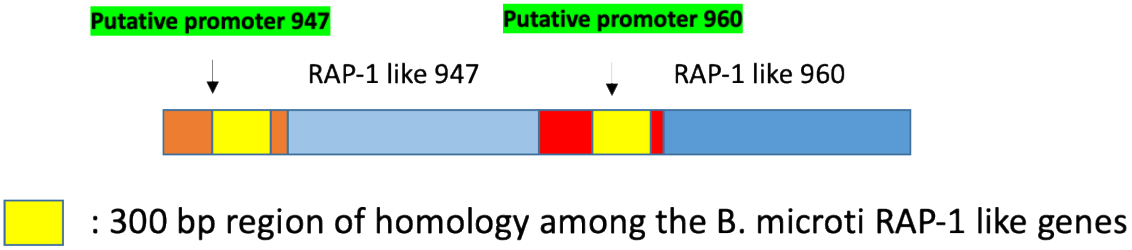


```
Query  689  ttttttAATATGAAGGAACGTATAGTAATATTAATATGCGATTTGCGGTATATTAGGTT  748
            ||||||||||||||||||||||||||||||||||||||||||||||||||||||||||||
Sbjct  395  TTTTTTAATATGAAGGAACGTATAGTAATATTAATATGCGATTTGCGGTATATTAGGTT  454

Query  749  TATGTGGCTGATTAGTGCAATGTAGTAATTGTCTATTG------------A----CCCAT  792
            ||||||||||||||||||||||||||||||||||||||            |    |||||
Sbjct  455  TATGTGGCTGATTAGTGCAATGTAGTAATTGTCTATTGTAGTAATTGTCTATTGGCCCAT  514

Query  793  GTGCCAATGCGTGTGTTATTACACGATTATACTTAATAATCGGATGAAACCAACACAAAA  852
            ||||||||||||||||||||||||||||||||||||||||||||||||||||||||||||
Sbjct  515  GTGCCAATGCGTGTGTTATTACACGATTATACTTAATAATCGGATGAAACCAACACAAAA  574

Query  853  GTTATCAATTTTGGAACGTGTGCAGTATGAATTAATTTATTCTTACACAATTTTATTCCT  912
            ||||||||||||||||||||||||||||||||||||||||||||||||||||||||||||
Sbjct  575  GTTATCAATTTTGGAACGTGTGCAGTATGAATTAATTTATTCTTACACAATTTTATTCCT  634

Query  913  TGTTGTTATGCCGATAGTTGCAGTGCACCCATTAGCCTAATTCTTAACTGTAAGGAATAC  972
            ||||||||||||||||||||||||||||||||||||||||||||||||||||||||||||
Sbjct  635  TGTTGTTATGCCGATAGTTGCAGTGCACCCATTAGCCTAATTCTTAACTGTAAGGAATAC  694

Query  973  ACTCACTC  980
            ||||||||
Sbjct  695  ACTCACTC  702
```

Figure S4. Schematic representation of the 300 bp region of homology among the *B. microti* RAP-1 like genes BMR1_03g00947 (947) and BMR1_03g00960 (960).

```
Pf  3752  NGDEYDGDEYDGDEYDGDECDGDEYDGDEYDGDEYDGDEYDGDEHNGDEYNGDEYNGDEY  3811
             NG+    G+    G+ ++G+    G+    G+ ++G+ +DG+    G+   +G+ +NG+ +NG+
Bm  353   NGEGSSGEGPSGEGFNGEGSSGEGPSGEGFNGEGFDGEGPSGEGPSGEGFNGEGFNGEGL  412

Pf  3812  NGDEYNGDEYNGDEYNGDEYNG  3833
             NG+   +G+   +G+   N   E+NG
Bm  413   NGEGPSGEGPSGEGLN--EWNG  432
```

Figure S5. Amino acid alignment between BmIPA48 (Bm) and PF3D7_1324300 (Pf). The only region of homology corresponds with a G-rich repeated region.

Phobius prediction-947

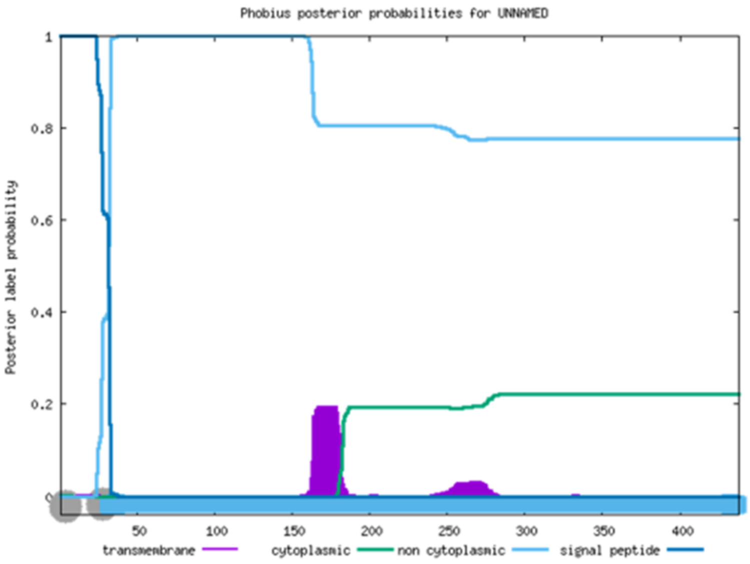

Figure S6. Secondary structure prediction of BmIPA48using the software Phoebius. Two putative transmembrane domains were confirmed by this predictive algorithm.
